# Expression levels of microRNAs are not associated with their regulatory activities

**DOI:** 10.1186/1745-6150-6-43

**Published:** 2011-09-19

**Authors:** Zhi Liang, Hong Zhou, Haoran Zheng, Jiarui Wu

**Affiliations:** 1 Hefei National Laboratory for Physical Sciences at Microscale and School of Life Science,, University of Science and Technology of China, Hefei, Anhui, China; 2 School of Computer Science and Technology, University of Science and Technology of China, Hefei, Anhui, China; 3 Key Laboratory of Systems Biology; Institute of Biochemistry and Cell Biology, Shanghai Institutes for Biological Sciences, Chinese Academy of Sciences, Shanghai, China

**Keywords:** MicroRNA, microRNA activity, microRNA expression

## Abstract

MicroRNAs (miRNAs) regulate their targets by triggering mRNA degradation or translational repression. The negative relationship between miRNAs and their targets suggests that the regulatory effect of a miRNA could be determined from the expression levels of its targets. Here, we investigated the relationship between miRNA activities determined by computational programs and miRNA expression levels by using data in which both mRNA and miRNA expression from the same samples were measured. We found that different from the intuitive expectation one might have, miRNA activity shows very weak correlation with miRNA expression, which indicates complex regulating mechanisms between miRNAs and their target genes.

**Reviewers:**

This manuscript was reviewed by an anonymous reviewer and Dr Yuriy Gusev.

## Findings

MicroRNAs (miRNAs) as small non-coding RNAs have been widely recognized as crucial regulators in a broad range of biological processes [1]. miRNAs regulate gene expressions by base-paring with the 3' untranslated region of target mRNAs, mainly leading to mRNA degradation or translational repression. Since one miRNA could regulate more than one target gene and one gene might be regulated by more than one miRNAs, the relationship between miRNAs and their target genes is quite complicated. Facing this challenge, several computational programs, including miReduce [2], MIR [3], Sylamer [4] and mirAct [5] have been developed to evaluate miRNA activity changes between different biological states according to gene-expression data. All these programs are based on a general consideration that an up-regulation of a miRNA's target expression should result from the decrease of its inhibitory activity, or vice versa. One could intuitively argue that the miRNA activities might be directly reflected by up- or down-regulation of miRNA expression levels. In order to test this argument, we investigated the relationship between miRNA activities determined by these four programs and miRNA expression levels by using data in which both mRNA and miRNA expression from the same samples were measured. Accordingly, two microarray data sets denoted as PNAS05 [6] and Nature05 [7] were analyzed. The data set PNAS05 contains matched expression data from 9 papillary thyroid carcinoma (PTC) samples and 9 unaffected thyroid tissue samples. The data set Nature05 contains matched mRNA and miRNA expression data from 8 different cancerous tissues and corresponding normal tissues.

**Figure 1:**
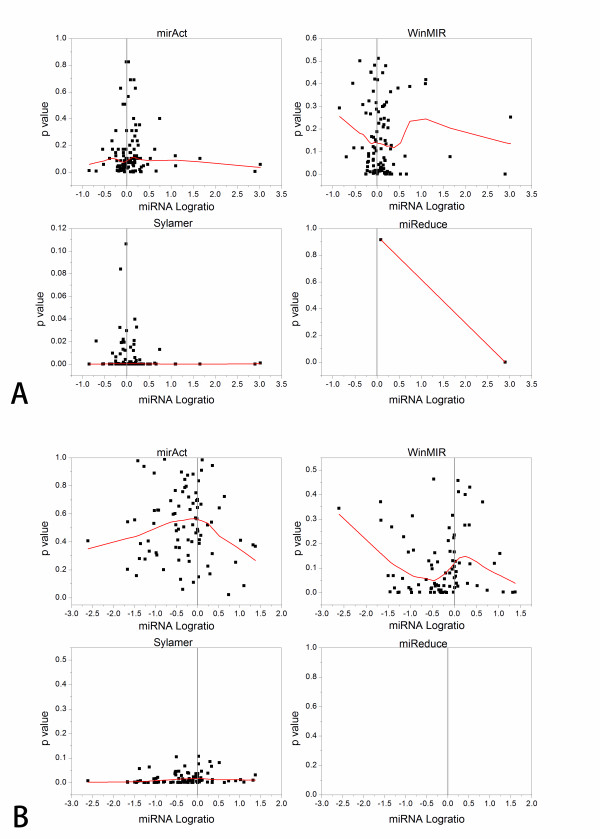
**The scatterplot of the data set PNAS2005 and Nature 2005 with four different programs**. (a) The × axis represents the log-ratio of miRNA levels in PTCs with respect to normal thyroid tissues based on the data set PNAS2005; (b) The × axis represents the log-ratio of miRNA levels in colon tumors with respect to corresponding normal tissues based on the data set Nature 2005. The y axis represents the raw *p*-value of each miRNA output by the programs. The regression line is generated using locally weighed scatterplot smoothing (LOWESS).

We assigned raw *p*-values calculated by the programs as indictors for miRNA activity change and log-ratios of miRNA expression levels in a tumor with respect to its corresponding normal tissue as indictors for miRNA expression changes. Figure 1 shows the scatterplot (*p*-value *vs*. log-ratio) and corresponding local regression curve for the data set PNAS05 and Nature05. Since a smaller *p*-value indicates a more significant activity change, we should expect an invert-V-like distribution of the points centered at the position with log-ratio equal to 0 if miRNA activity is closely correlated with miRNA expression. However, we didn't observe such phenomena (Figure 1 and Additional File 1, Figures S1-S7). To give a quantitative evaluation of the fitness of miRNA activity-expression relationship to an invert-V shape, we calculated the correlation coefficient between the raw *p*-values and the absolute of miRNA log-ratios. As shown in Table 1, there were very weak correlations between the raw *p*-values and the absolute of miRNA log-ratios in both data sets. Taken together, these results indicate that the levels of miRNA expression are not always associated with their activities, which is in agreement with previous observations that an increase in activity of miRNAs in PTC and breast cancer was not accompanied by global increase in the levels of miRNAs [8,9]. A recent paper has also shown that the overall correlation between miRNA activity and expression is very weak in prostate cancer [10].

**Table 1:** Correlation coefficients between the raw *p*-values and the absolute of miRNA log-ratios

	mirAct	WinMIR	Sylamer	miReduce*
**PNAS2005**	-0.1612	0.0505	-0.1021	-1
**Nature2005**				
COLON	-0.1948	0.0174	-0.2136	-
KID	-0.0586	0.1178	-0.1341	-
BLDR	0.0566	0.1176	0.0651	-
PROST	-0.103	0.0346	-0.1681	-
LUNG	-0.0904	-0.0371	0.1925	-
BRST	-0.1096	-0.165	-0.0076	-
PAN	0.133984	-0.01169	-0.05647	-
UT	-0.0954	0.0091	-0.1221	-

* Few miRNAs (only one or two; see Figure 1 and Additional File 1, Figures S1-S7) were detected by miReduce in all the data sets. So most cells of this column were blank and the -1 in PNAS2005 data set was due to the fact that only two miRNAs were detected.

The present results suggest that the regulatory activity of miRNAs should depend on many different factors besides their expression levels. It has been shown that RNA accessory proteins such as RNA binding proteins (RBPs) can regulate miRNA activity without impacting miRNA expression levels and blur the relationship between miRNA activity and miRNA expression [11]. For instance, a human-RBP Dnd1 regulated miRNA activity by binding mRNAs and determining access for miRNA regulators [12]. Similarly, in *E. coli*, a protein Hfq was found to bind to mRNAs and determines sRNA activity [13]. Furthermore, it was demonstrated that miRNA activity is also influenced by target abundance [14]. Therefore, these discovered various factors impacting miRNA activity make the situation more complex.

In summary, different from the intuitive expectation one might have, miRNA activity shows very weak correlation with miRNA expression, which indicates complex regulating mechanisms between miRNAs and their target genes.

## List of abbreviations

microRNA: miRNA.

## Competing interests

The authors declare that they have no competing interests.

## Authors' contributions

ZL and HZ performed the analysis. ZL, HZ, HRZ and JRW wrote the manuscript. All authors read and approved the final manuscript.

## Reviewers' comments

### Reviewer's report 1

Using a computer program generated p-value as an indicator of miRNA regulatory activity and analysis of correlation, the authors conclude that the association between expression levels of miRNAs and their regulatory activities is very weak. One could argue that it is possible to reach the same conclusion by considering available evidences alone without having to rely on the statistical (correlation) analysis of two variables as done in this article. It should be noted that although p-values are generated by computer programs that analyze activity change of miRNAs, it is not clear what these values actually represent: either biologically or statistically. Biologically, it would be more meaningful to focus analyses only on those miRNAs that have similar regulatory activities, rather than lumping together all miRNAs in one analysis. It is also statistically problematic because the analyses of simple correlation failed to consider potential confounding factors such as age and sex for tissues that provide data for computer programs and the resultant p-values. The importance of considering confounding factors in this type of data analysis has been articulated in Potter (2003)* Therefore, the conclusion from the analysis should be considered superficial, and unnecessary.

*Potter J. Epidemiology, cancer genetics and microarrays: making correct inferences, using appropriate designs. Trends Genet 2003, 19(12):690-695.

### Author response

Dear reviewer, thank you for your careful reviewing on our manuscript. The following are our responses to your comments.

First, the statistical analysis performed in the manuscript aims to identify the general relationship between miRNA activity and miRNA expression. Although some available evidences support the absence of association between the two, however some support the opposite. Also, current evidences are limited and most of them are case-by-case examples. So we think it might not be appropriate to make a general conclusion by considering available evidences alone, at least at the present time. Especially, it is difficult to measure the biological activity of a miRNA via direct experimental techniques. One effective and generally accepted alternative is to evaluate activity of a miRNA by assessing the expression of its targets. The computational determined activities make it possible to systematically evaluate the association between miRNA expression levels and biological activities.

Second, the p-values generated by the programs reflect the significance of miRNA activity changes. In fact, all the scoring systems of the four computational programs used in the manuscript have explicit definition and meaning. For instance, the mirAct web server infers the regulatory effect of a miRNA via a two-step procedure. First, a sample score measuring the activity of a miRNA in a sample is obtained by comparing the expression levels of its non-targets with those of targets. In the case of rank transformation of expression values, the difference of the average ranks between a miRNA's non-targets and targets is used. In the case of z-score representation of expression values, the two-sample t-statistic is applied. Then, the miRNA activity changes across different classes of samples are investigated by examining the sample scores via Kruskal-Wallis test, which tests the null hypothesis that all classes have identical miRNA activity.

Third, we highly appreciate your preciseness on the confounding factors that might influence data analysis. We completely agree your opinion that care must be taken when dealing with high-throughput data. That's why we performed the analysis using expression data from nine different tissues and four different computational programs. We think our results should be reliable. On one hand, as stated by the data providers that they had taken strategies to control the confounding factors that might influence their experiments. On the other hand, we repeated the analysis using different tissues and programs and obtained stable results.

Reviewer, Dr Yuriy Gusev, Georgetown University Medical Center, United States of America

This reviewer provided no comments for publication.

## Supplementary Material

Additional file 1 **Supplementary text and figures**. Click here for file
