# Supplementary material for: Expression levels of microRNAs are not associated with their regulatory activities
Source: Biol Direct. 2011 Sep 19;6:43. doi: 10.1186/1745-6150-6-43 (PMC3189187; doi:10.1186/1745-6150-6-43)
Supplement: Additional file 1 — Supplementary text and figures. [file 1745-6150-6-43-S1.DOC]

**Supplementary Data**

Supplementary Text …………………………………………………………………….1

Figures S1-S7………………………………………………………………2

**Supplementary Text**

(1) Expression data

Two data sets Nature05 (Lu et al., 2005) and PNAS05 (He et al., 2005) were used in this study. The normalized mRNA and miRNA expression data of Nature05 was downloaded from the accompanied web site of the paper (http://www.broad.mit.edu/cancer/pub/migcm). The normalized mRNA expression data of PNAS05 was downloaded from NCBI GEO (GSE3467). The raw data of miRNA expression data of PNAS05 was download from ArrayExpress (E-TABM-68) and the normalization was performed by following the instructions described in (He et al., 2005) with BRB ArrayTools (Richard Simon and Amy Peng Lam, National Cancer Institute, Bethesda).

(2) The regulation relationships between miRNAs and genes

TargetScan 5.1 (Lewis et al., 2005) predictions of miRNA-target regulation relations were used in the analysis. For mirAct (Liang et al., 2011), it is straightforward by choosing the target predictions as TargetScan 5.1. Declaration of miRNA-gene relationships for MIR (Cheng et al., 2008) is achieved by specifying a binary relation matrix with genes as rows and miRNAs as columns, in which ones represent TargetScan miRNA-gene regulations. For miReduce (Sood et al., 2006) and Sylamer (van Dongen et al., 2008) which require 3’ UTRs of genes as input and report motifs enriched in up- and down-regulated genes, the 3’ UTR sequences of genes downloaded from the TargetScan web site were used and the miRNA-gene regulation relationships were determined by checking a motif for the presence of a corresponding TargetScan miRNA seed.

**REFERENCES**

Cheng, C. and Li, L.M. (2008) Inferring microRNA activities by combining gene expression with microRNA target prediction. *PLoS One*, **3**, e1989.

He, H., Jazdzewski, K., Li, W., et al. (2005) The role of microRNA genes in papillary thyroid carcinoma. *Proc Natl Acad Sci USA*, **102**, 19075-19080.

Lewis, B. P., Burge, C. B., Bartel, D. P. (2005) Conserved seed pairing, often flanked by adenosines, indicates that thousands of human genes are microRNA targets. *Cell*, **120**, 15–20.

Liang, Z., Zhou, H., He, Z., et al. (2011) mirAct: a web tool for evaluating microRNA activity based on gene expression data. Nucleic Acids Res, accepted

Lu, J., Getz, G, Miska, E.A., et al. (2005) MicroRNA expression profiles classify human cancers. *Nature*, **435**, 834-838.

Sood, P., Krek, A., Zavolan, M., et al. (2006) Cell-type-specific signatures of microRNA on target mRNA expression. *Proc*. *Natl*. *Acad*. *Sci*. *U. S. A.*, **103**, 2746-2751.

van Dongen, S., Abreu-Goodger, C. and Enright, A.J. (2008) Detecting microRNA binding and siRNA off-target effects from expression data. *Nature Methods*, **5**, 1023-1025.


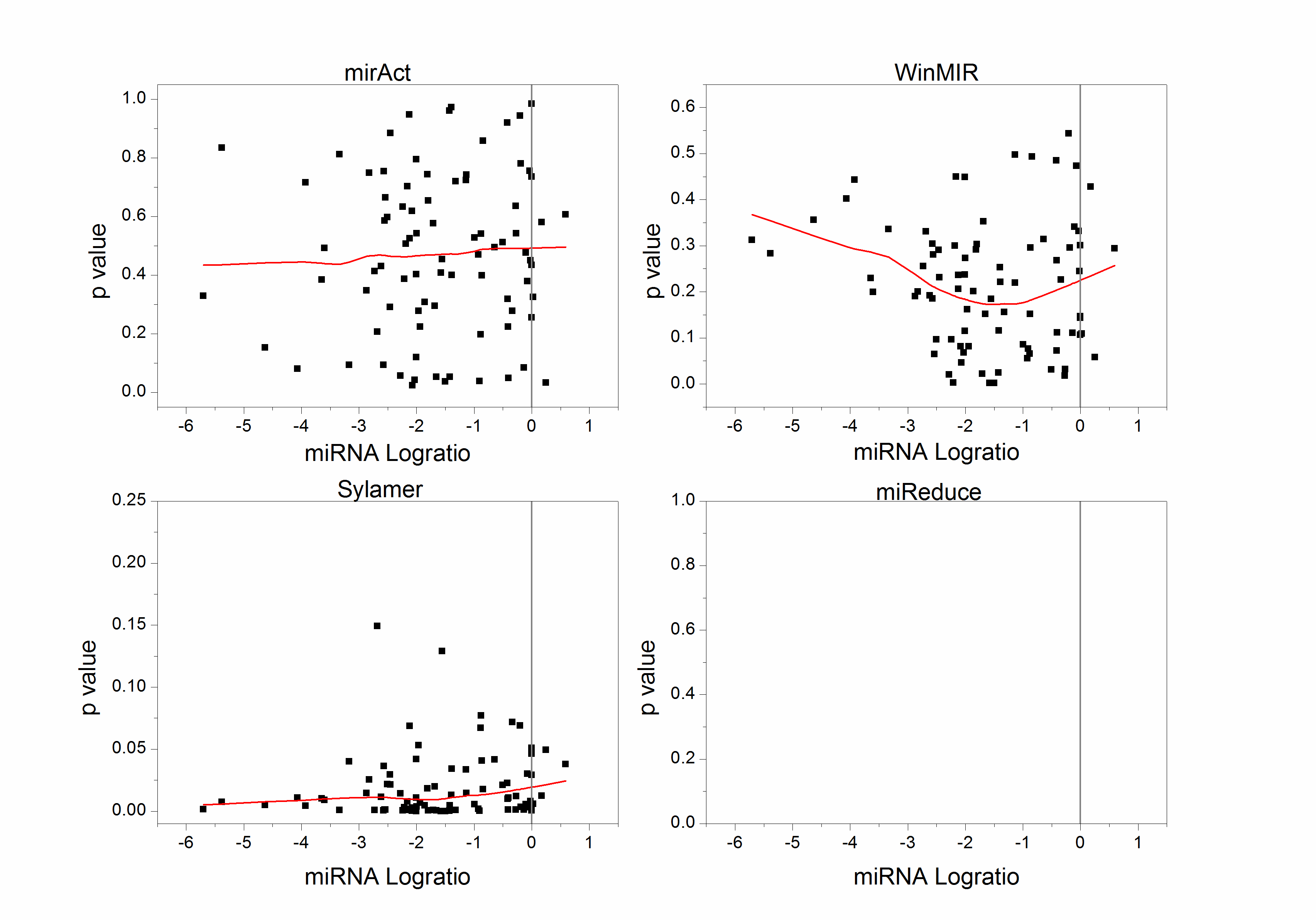


Figure S1. The scatterplot of the data set Nature2005 (KID). The x coordinate is the log-ratio of miRNA levels in kidney tumors with respect to corresponding normal tissues, the y coordinate is the raw p value of each miRNA output by different programs. The red line is generated using locally weighed scatterplot smoothing (LOWESS).


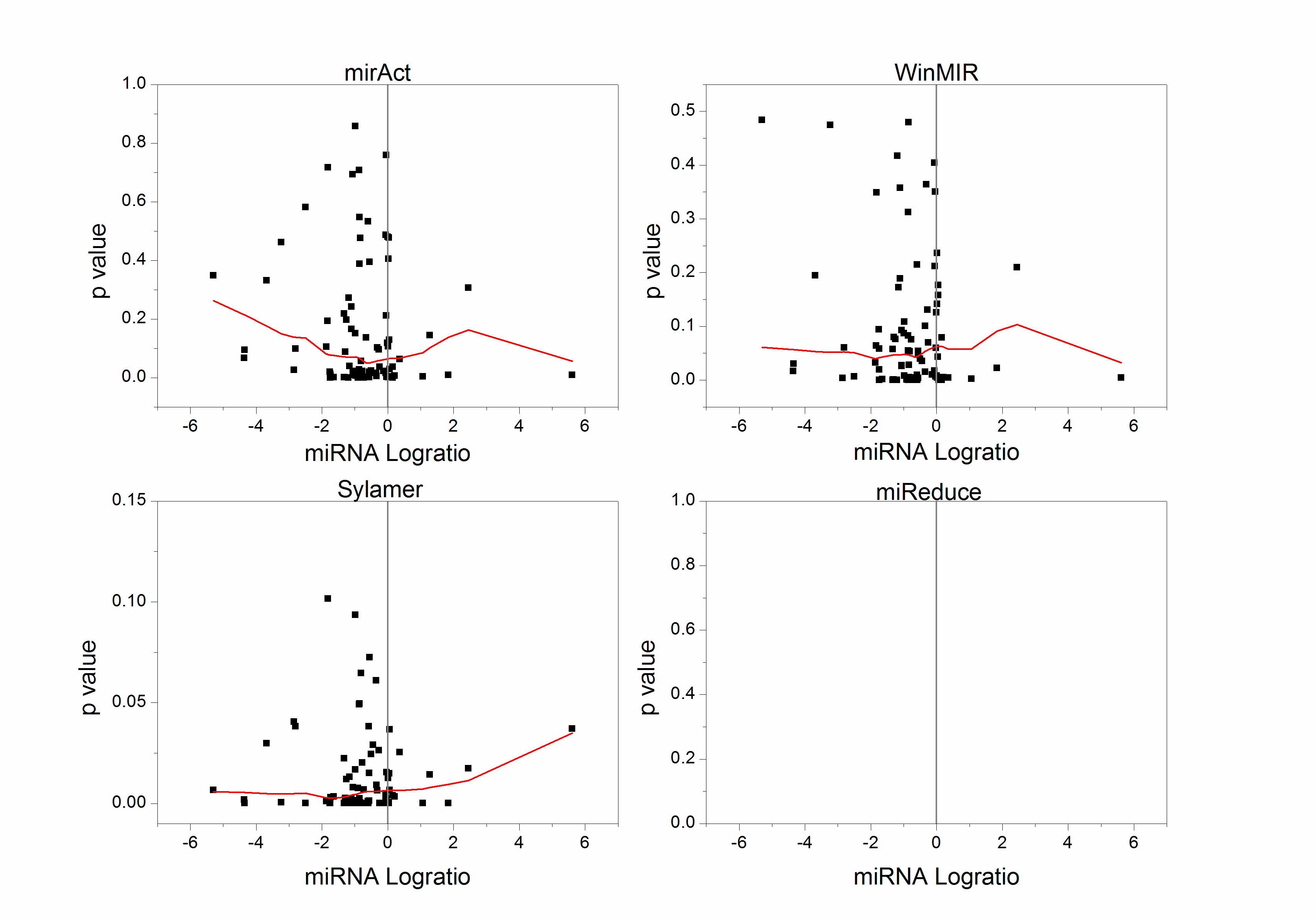


Figure S2. The scatterplot of the data set Nature2005 (BLDR). The x coordinate is the log-ratio of miRNA levels in bladder tumors with respect to corresponding normal tissues, the y coordinate is the raw *p*-value of each miRNA output by the programs. The red line is generated using locally weighed scatterplot smoothing (LOWESS).


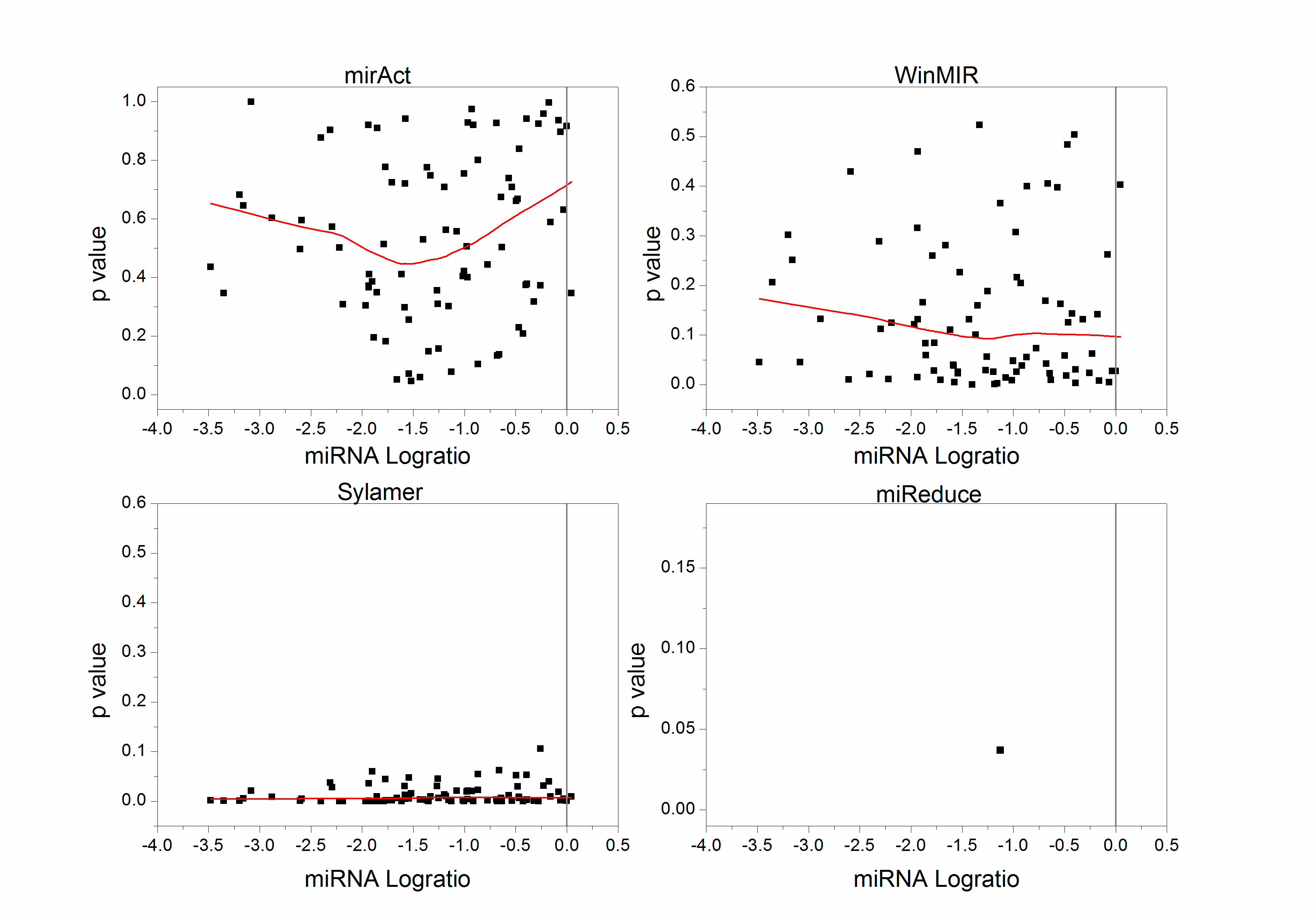


Figure S3. The scatterplot of the data set Nature2005 (PROST). The x coordinate is the log-ratio of miRNA levels in prostate tumors with respect to corresponding normal tissues, the y coordinate is the raw *p*-value of each miRNA output by the programs. The red line is generated using locally weighed scatterplot smoothing (LOWESS).


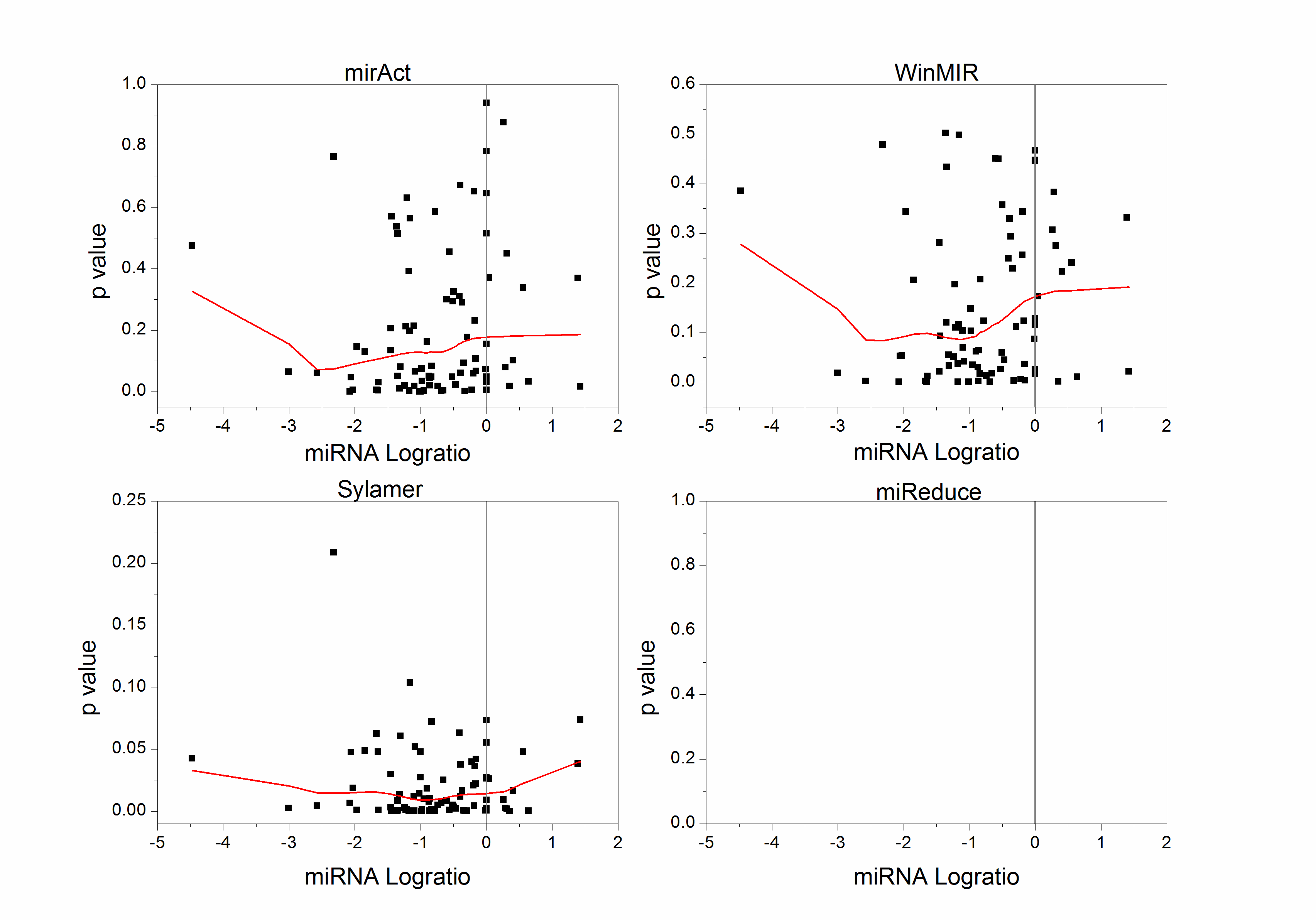


Figure S4. The scatterplot of the data set Nature2005 (LUNG). The x coordinate is the log-ratio of miRNA levels in lung cancers with respect to corresponding normal tissue, the y coordinate is the raw *p*-value of each miRNA output by the programs. The red line is generated using locally weighed scatterplot smoothing (LOWESS).


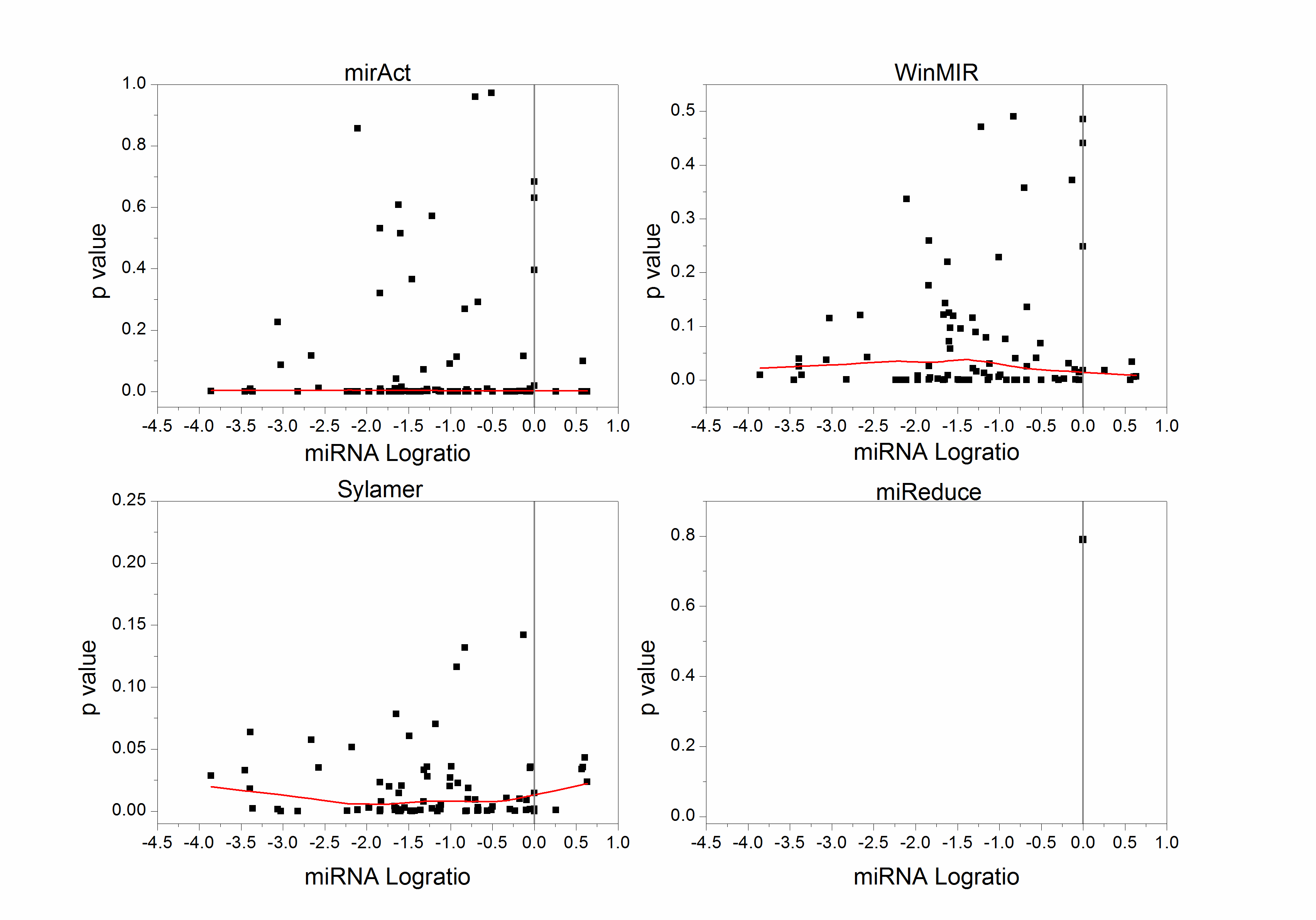


Figure S5. The scatterplot of the data set Nature2005 (BRST). The x coordinate is the log-ratio of miRNA levels in breast cancers with respect to corresponding normal tissues, the y coordinate is the raw *p*-value of each miRNA output by the programs. The red line is generated using locally weighed scatterplot smoothing (LOWESS).


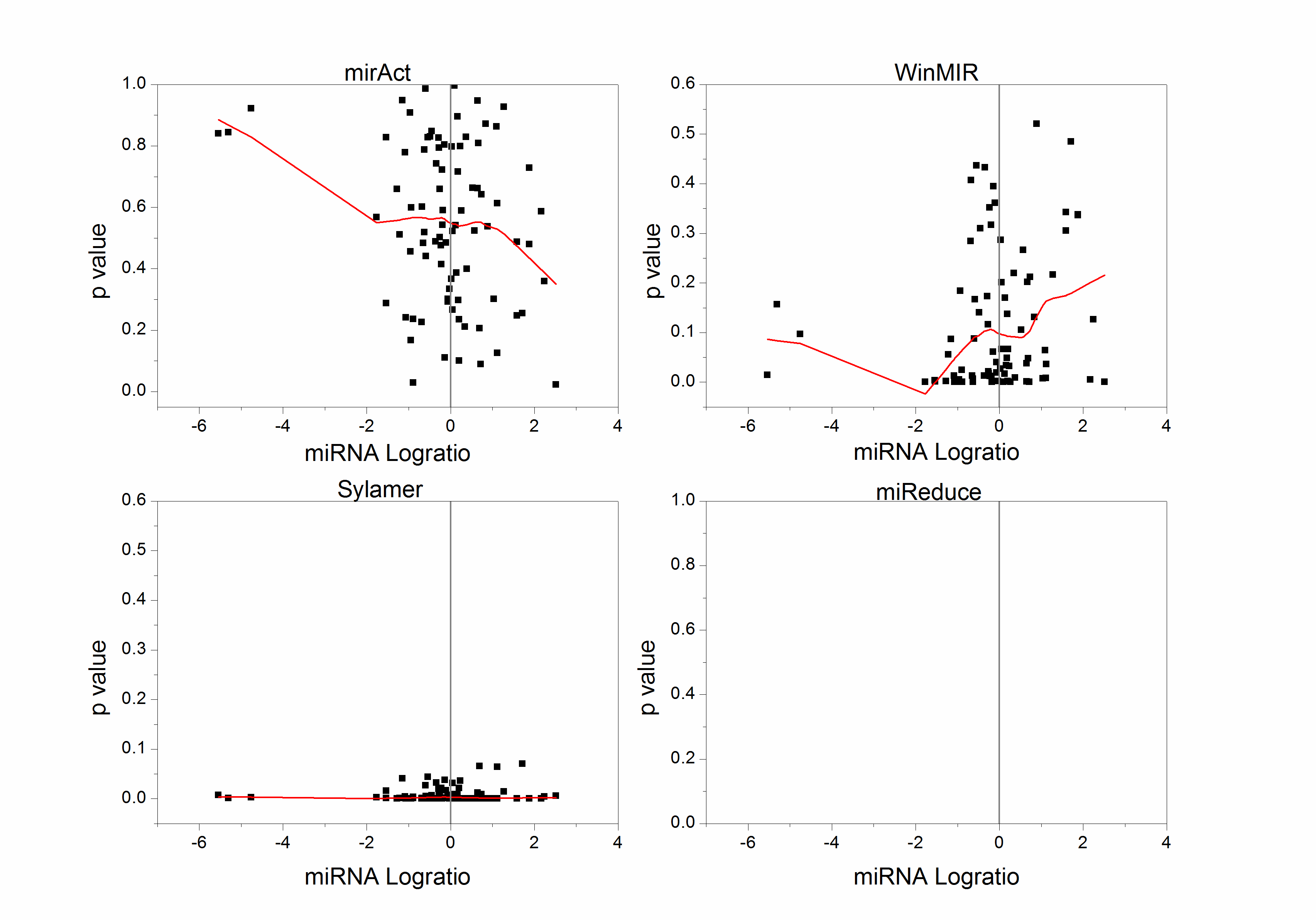


Figure S6. The scatterplot of the data set Nature2005 (PAN). The x coordinate is the log-ratio of miRNA levels in pancreas cancers with respect to corresponding normal tissues, the y coordinate is the raw *p*-value of each miRNA output by the programs. The red line is generated using locally weighed scatterplot smoothing (LOWESS).


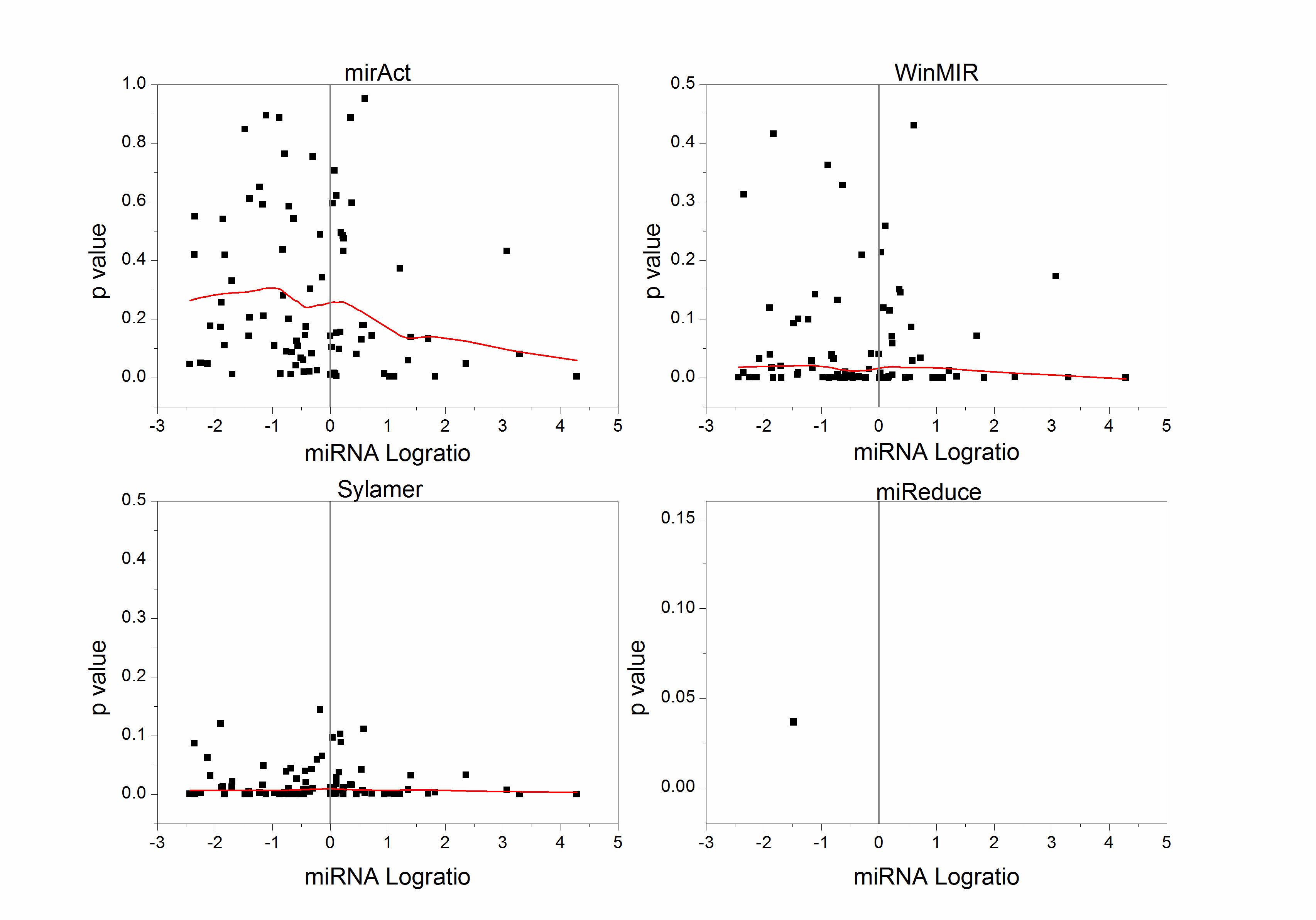


Figure S7. The scatterplot of the data set Nature2005 (UT). The x coordinate is the log-ratio of miRNA levels in uterus cancers with respect to corresponding normal tissues, the y coordinate is the raw *p*-value of each miRNA output by the programs. The red line is generated using locally weighed scatterplot smoothing (LOWESS).
